# Unravelling the contribution of early postseismic deformation using sub-daily GNSS positioning

**DOI:** 10.1038/s41598-019-39038-z

**Published:** 2019-02-11

**Authors:** Cedric Twardzik, Mathilde Vergnolle, Anthony Sladen, Antonio Avallone

**Affiliations:** 1 Université Côte d’Azur, CNRS, Observatoire de la Côte d’Azur, IRD, Geoazur, UMR 7329, 250 rue Albert Einstein, Sophia-Antipolis, 06560 Valbonne, France; 2 Istituto Nazionale di Geofisica e Vulcanologia, Centro Nazionale Terremoti, Via di Vigna Murata 605, Rome, 00143 Italy

## Abstract

After large earthquakes, parts of the fault continue to slip for days to months during the afterslip phase, a behaviour documented for many earthquakes. Yet, little is known about the early stage, i.e., from minutes to hours after the mainshock. Its detailed study requires continuous high-rate position time series close to the fault, and advanced signal processing to accurately extract the surface displacements. Here, we use refined kinematic precise point positioning processing to document the early postseismic deformation for three earthquakes along the South American subduction zone (2010 M_*w*_8.8 Maule, Chile; 2015 M_*w*_8.3 Illapel, Chile; 2016 M_*w*_7.6 Pedernales, Ecuador). First, we show that early afterslip generates significant surface displacement as early as a few tens of minutes after the earthquake. Our analysis of the time series indicates that, over the first 36 hours, more than half of the displacement occurs within the first 12 hours, a time window often disregarded with daily positioning. Thus, estimates of coseismic offsets can be biased by more than 10% if early postseismic displacements are acknowledged as coseismic ones. Finally, these results highlight the difficulty to accurately evaluate the different contribution to the seismic cycle budget and thus the associated hazard on faults.

## Introduction

The postseismic phase marks the transition between the earthquake coseismic rupture and the interseismic phase, when the fault is re-locking. It was first documented in the early 1950s after the 1946 Nankaido, Japan earthquake (M_*w*_8.1)^1,2^. In the mid to late 1960s additional observations were made after the 1964, Niigita, Japan, earthquake (M_*w*_7.6)^3^, and the 1966 Parkfield, California, earthquake (M_*w*_6.0)^4^. With the advances of satellite geodesy in the 1990s, the number of observations has increased considerably, and the postseismic phase is now the focus of many studies (see for instance the data compilation from Ingleby and Wright)^5^.

The term postseismic encompasses different processes occurring as a response of the earthquake rupture such as poroelastic and viscoelastic relaxation, or transient aseismic slip on the fault, called afterslip. In this study, we focus in particular on afterslip, which might hold some answers to several relevant questions about the physical properties of faults. First, recovering the spatial distribution of afterslip allows to document the different styles of slip^6^, constrain the level and scale of frictional heterogeneities, as well as the physical conditions driving slip. Afterslip also represents a large fraction of the total slip budget of a fault. Indeed, the amount of postseismic slip can sometimes exceed the coseismic slip after a few months or years. For instance, this is observed for the M_*w*_7.7 1994 Sanriku-Haruka-Oki, Japan earthquake^7^ and the M_*w*_6.0 2004 Parkfield, California, earthquake^8^. For these two earthquakes, equivalent moment magnitude of the early postseismic slip (i.e., 5 days and 1 day, respectively) is 30 and 50% greater than the coseismic moment magnitude^7,9^. Finally, many studies suggest that afterslip might be a controlling mechanism of aftershocks as both phenomena directly follow the mainshock and show a similar temporal evolution *e*.*g*.^10–13^.

Most postseismic studies model afterslip using rate-and-state friction, a formalism first introduced by Dieterich^14^, on the basis of observations from laboratory experiments. These studies show that the surface displacement induced by afterslip can be explained under this framework *e*.*g*.^11,12,15–18^. However, the starting phase (<1 day) is critical to better understand the mechanics of afterslip. Wennerberg and Sharp^11^ have attempted to explain the surface displacement observed for several earthquakes using the rate-and-state friction law, as well as the rate-dependent friction law, a widely used variant assuming steady-state. Even though both models are able to explain the surface observations, they show that when the models are extrapolated towards the origin time of the earthquake, the two start to diverge. This is also pointed out by Helmstetter and Shaw^19^, who have extended the comparison to the rate-and-state friction law under velocity-strengthening (i.e., stable aseismic slip) and velocity-weakening (i.e., unstable slip) regime, the rate-dependent friction law, and an empirical law based on the observed time decay of aftershocks. This discrepancy at the early stage of the postseismic phase has been explained based on a theoretical approach^20^. It is due to the response of a fault to a sudden stress perturbation,which follows two stages: (1) an initial acceleration of afterslip over a given time (*t*_*max*_) up to a peak velocity, followed by (2) a long-term steady-state relaxation. Thus, the steady-state approximation that is generally used to model afterslip is only valid after a certain time (*t*_*max*_), which is estimated to range from 10^−6^ seconds up to 2 days^20^. This latter result calls for more observations to precisely document what happens during the time frame from few seconds to few days after an earthquake, hereafter called the early postseismic phase. However, to this date, few observations are available.

Langbein *et al*.^21^ are among the first to investigate the early postseismic phase. They used sub-daily GNSS position time series at 13 sites, with variable positioning intervals (1-minute, 3-minute, and 30-minute) to capture the time evolution of surface displacement after the 2004 Parkfield earthquake, as early as 100 seconds and up to 10 days after the mainshock. After that, they used daily GNSS time series up to 9 months following the earthquake. They show that the entire time series at all sites can be explained by an Omori’s-type friction law, as what is typically used to explain the behaviour of aftershocks^22^. Miyazaki and Larson^23^ went a step further by performing a spatiotemporal inversion of afterslip after the 2003 Tokachi-Oki, Japan, earthquake (M_*w*_8.0). They used 30-second GNSS kinematic position time series that cover the first 4 hours following the mainshock. The surface observations show very little displacement for the first hour, followed by an intensification of the displacement for the next three hours, which results in a complex pattern of afterslip on the fault. For the first hour, and preceding the occurrence of a large aftershock (M_*w*_7.4), afterslip reaches ~3 cm of peak slip, and is located in between the rupture areas of the mainshock and of the aftershock. In the next three hours, a second slip patch is observed, down-dip of the rupture area of the mainshock, with a peak slip of ~12 cm. According to Miyazaki and Larson^23^, the mainshock triggers afterslip next to its rupture area, leading to the occurrence of the large aftershock one hour later, which then triggers the down-dip afterslip patch. However, Fukuda *et al*.^24^ show that the same surface observations can also be explained using the rate-and-state framework alone, without involving the occurrence of a large aftershock. Their interpretation is that the small surface displacement observed for the first hour reflects the acceleration phase predicted by Perfettini and Ampuero^20^, before that afterslip switches to the steady-state regime. Later, Malservisi *et al*.^25^ looked at the early postseismic displacement (i.e., the first day) after the 2012 Nicoya, Costa Rica, earthquake (M_*w*_7.6). They show that the signal is immediately intense, and it decays very rapidly with time. Thus, for this earthquake, it suggests that the steady-state regime is set almost immediately after the mainshock. In addition, while this earthquake has a smaller magnitude than the Tokachi-Oki earthquake, the inverted peak slip amplitude of the early afterslip is about two times larger (~30 cm instead of ~12 cm). Finally, on a similar time scale (4 hours), Munekane^26^ looked at the 2011 Tohoku-Oki, Japan, earthquake (M_*w*_9.1). Here, after 1 hour, afterslip reached an equivalent moment magnitude of 7.8 and a peak slip of ~21 cm. Interestingly, this is about 30% less than for the Nicoya earthquake. Thus, early afterslip might not necessarily scale with the magnitude of the mainshock, as observed at the time scale of a few months^27^.

The diversity of results regarding the early postseismic phase, whether in terms of frictional properties, slip amplitude, or temporal evolution, stresses the need to better document this phase of the seismic cycle. Early afterslip is essential to understand how faults transition from coseismic fast slip to postseismic slow slip, both in space and time, and to refine its contribution to the total postseismic slip budget. In particular, considering that the amplitude of afterslip tends to decay exponentially with time, we can expect that the early postseismic displacement is significant. This is why, in this study, our main goal is to provide new observations of the surface displacements generated during the early postseismic phase.

**Figure 1:**
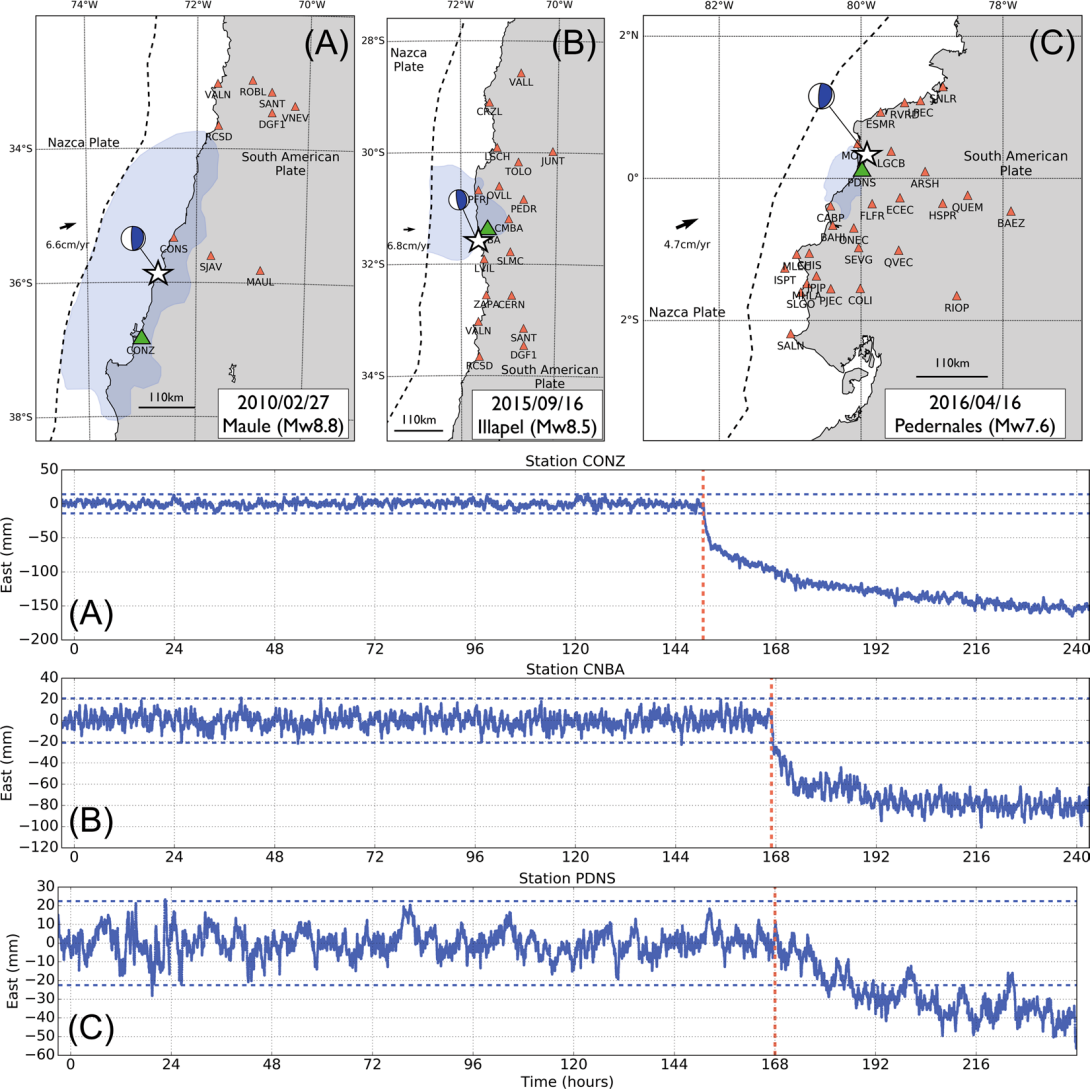
(Top row): Map showing the distribution of continuous GNSS stations for each earthquake considered in this study. There are 10, 17, and 26 stations for the Maule, Illapel, and Pedernales earthquakes, respectively. The continuous GNSS stations for the Maule and Illapel earthquakes are part of the International GNSS Service (IGS) and the Chilean-French International Laboratory (LIA) networks. The stations for the Pedernales earthquake are part of the IGEPN (Instituto Geofísico) and IRD (Institut de Recherche pour le Développement) network. The hypocenters (white stars) are retrieved from the Global Centroid Moment Tensor catalog (http://www.globalcmt.org, last accessed on December 2018). The blue shaded areas show the areas of coseismic slip for each earthquake, as inferred by Vigny *et al*.^56^, Ruiz *et al*.^57^ and Nocquet *et al*.^58^. (**A**) East position time series for the station CONZ for the 2010 Maule, earthquake. (**B**) East position time series for the station CNBA for the 2015 Illapel, earthquake. (**C**) East position time series for the station PDNS for the 2016 Pedernales, earthquake. For the three time series, the red dashed lines show the earthquake origin time and the blue dashed lines show the mean of the time series before the earthquake plus and minus 3.3 times the standard deviation of the time series, also calculated before the earthquake. The location of the stations is shown by green triangles on the maps.

Because of the short time scale of the early postseismic phase (few hours), we cannot use accurate daily GNSS position time series. Instead, we need to work on continuous sub-daily, high-frequency, GNSS position time series. These time series contain more noise and require the use of advanced kinematic processing and analysis techniques to isolate the emerging signal from the noise as early as possible. Here, we use a kinematic precise point positioning multi-stage strategy to process the 30-second continuous GNSS data, an adapted sidereal filter to refine the position time series, and a statistical detection test to extract the early postseismic displacement following 3 megathrust earthquakes in South America: the 2010 Maule, Chile, earthquake (M_*w*_8.8), the 2015 Illapel, Chile, earthquake (M_*w*_8.3), and the 2016 Pedernales, Ecuador, earthquake (M_*w*_7.6). Figure 1 shows the network of stations that we use for each earthquake. We have chosen these three  earthquakes because of their relative proximity to the coast, maximising the chances to observe significant signal over the first few hours. With our processing and post-processing routines detailed in the Method Section, we obtain 3-component 30-second position time series over 10 days (6 days before the earthquake, the day of the earthquake itself, and 3 days after the earthquake) for a total of 53 stations. Hereafter, we only focus on the East component, which is the one that typically records the largest motion during and after large megathrust earthquakes on the South American subduction zone. Figure 1 (ABC) show a sample of the time series that are representative of the data set that we analyse in this study. These observations are used to evaluate how quickly afterslip generates a detectable signal at the surface, and to document how intense is the early postseismic surface displacement. We then discuss the implications of our results on the estimation of the earthquake cycle slip budget.

## Results

### Early postseismic displacement greatly affects coseismic offset estimates

Before analysing our observations with a focus on the postseismic phase only, we have first attempted to quantify the impact of early postseismic surface displacements on the estimation of coseismic offsets, a question often raised during the retrieval of coseismic slip distributions using geodetic data *e*.*g*.^28–30^.

Our strategy (see the Method Section) consists of processing separately the data before the earthquake (up to 30 seconds before the earthquake origin time) and after the earthquake (from 5 minutes after the earthquake). Thus, the coseismic offsets that we calculate from our time series should only be affected by very early postseismic displacement. On the contrary, a significant number of studies use daily position time series to estimate the coseismic offsets, and the strategy used for the calculations varies. For instance, Lorito *et al*.^31^ use 7 to 8 days before the Maule earthquake to compute the pre-earthquake position and the position on the day of the earthquake for the post-earthquake position. Meanwhile, Ding *et al*.^32^ use 4 days before the earthquake and 4 days after the earthquake to compute the coseismic offsets of the 2013 Craig, Alaska, earthquake (M_*w*_7.5). A similar approach is used for the 2012, Haida Gwai, Canada, earthquake (M_*w*_7.8), except that 7 days are used before and after the earthquake^33^. Thus, it is possible that some postseismic displacement contaminates these coseismic offset estimates.

Langbein *et al*.^9^ attempted to quantify this effect for the 2004 Parkfield earthquake. They show that depending on the rate of positioning (1-minute, 30-minute, 1-day), the estimated coseismic offsets can differ by a few millimetres. Hill *et al*.^34^ did a similar study after the 2012 Mentawai, Indonesia, earthquake (M_*w*_7.8). They compared the coseismic offsets estimated using daily position time series and those estimated using 1-second position time series. For the former, 8 days before and after the earthquake is used, while 90 seconds of data on either side of the earthquake is used for the 1-second position time series, with the first 2 minutes following the earthquake origin time being avoided. They show that the coseismic offsets with the 1-second position time series are smaller than the estimates from daily positions time series. In fact, they estimate that ~30% of the offsets measured using daily position time series is not caused by the earthquake but by afterslip.

**Figure 2:**
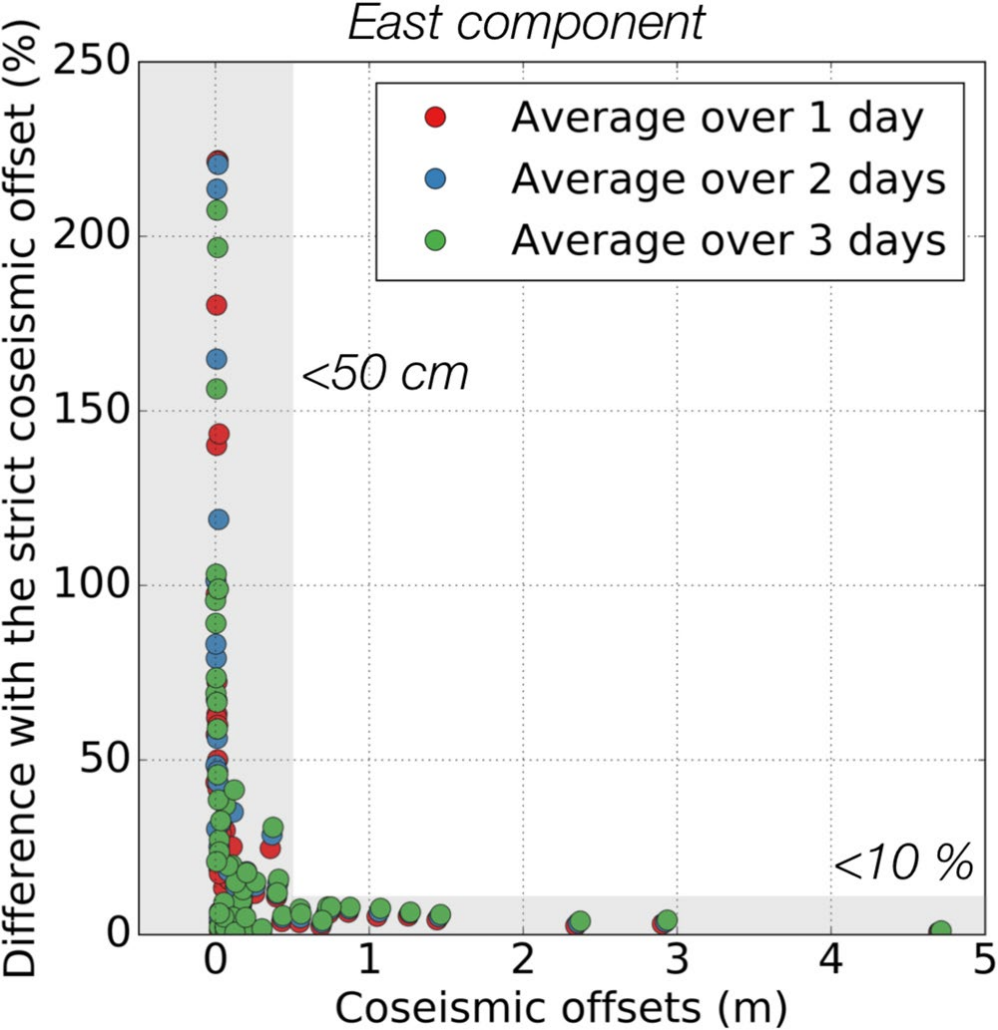
Strict coseismic offset estimations versus offsets determined using an average of the position time series over 24 hours (red dots), 48 hours (blue dots) and 72 hours (green dots). The horizontal shaded area shows the region where the different estimates do not differ by more than 10%. The vertical shaded area shows the region where the estimates differ by more than 10%, when the measured offsets are less than 50 cm. The dataset used for this Figure is available in Section C of the Supplementary Materials.

Here, we further quantify the bias from including postseismic displacement in estimates of coseismic offsets. To do so, we compare the strict coseismic offsets calculated from the 30-second position time series, with offsets calculated by averaging the position time series over one, two and three days before and after the earthquake. We find that the difference between the strict coseismic offsets and those estimated using averages over one or several days is ~34% (see Fig. 2). This is consistent with what has been determined for the Mentawai earthquake^34^. As expected, when the offsets are large (> 50 cm), early postseismic displacement is negligible compared to the measured offset (<10%). On the contrary, Fig. 2 shows that care must be taken when dealing with small coseismic offsets. In that case, early postseismic displacement starts being significant compared to the measured offset (up to 200%).

### Postseismic displacement can be observed within tens of minutes after an earthquake

To observe postseismic displacement at the surface, enough slip on the fault should be accumulated in order for the signal to rise above the noise level. Here, we attempt to quantify the duration of this process, i.e., how long it takes for the afterslip to generate a detectable displacement at the surface. This provides important information regarding how rapid early afterslip is. For instance, a late detection might suggest a slow initiation while an early detection might point towards a rapid start of the afterslip. However, prior to the detection of significant surface displacement, we might not be able to distinguish between a mechanical model in which afterslip has not yet generated detectable surface displacement, and a mechanical model where the start of afterslip has simply been delayed.

**Figure 3:**
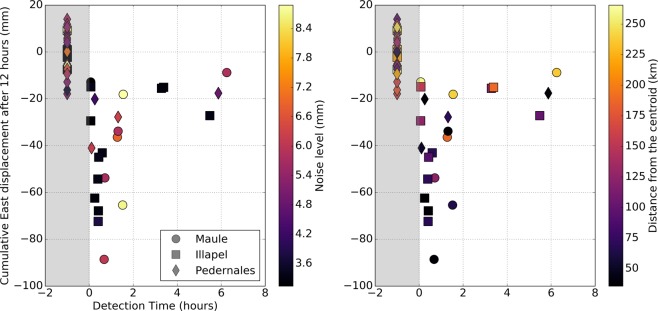
Figure showing the onset time of the postseismic displacement versus the amplitude of the postseismic displacement measured 12 hours after the earthquake, on the East component. Each point is color-coded with respect to the noise level of the time series on the left side (i.e., the standard deviation of the time series calculated over the 6 days before the earthquake), and with respect to the distance from the centroid on the right side. The onset of postseismic displacement could be detected for 23 stations out of 53 analysed time series (see Table 1). The shape of the symbols corresponds to a given earthquake (see the lower right inset). All the stations that failed the detection test are shown inside the grey area at a fake detection time (−1 hour). The data used to produce this figure are shown in Section D of the Supplementary Materials.

**Table 1:** Statistics about the detection of the onset time of postseismic surface displacement. The entire dataset is available in Section D of the Supplementary Materials.

	Number of detection	Mean onset time	Median onset time
2010 Maule earthquake (M_*w*_8.8)	8/10 (80%)	1.7 ± 1.8 hours	1.3 hours
2015 Illapel earthquake (M_*w*_8.3)	11/17 (65%)	1.3 ± 1.4 hours	0.4 hours
2016 Pedernales earthquake (M_*w*_8.8)	4/26 (15%)	1.8 ± 2.3 hours	0.8 hours
Overall	23/53 (43%)	1.5 ± 1.9 hours	0.7 hours

To address this question, we apply the Chow test^35^ in order to detect the presence of a structural break in the time series, i.e., a significant change in the mean. We adapt the test so that the time of the structural break or onset time (t_*onset*_), for a given receiver is the time when the mean of the time series has changed by more than 3 times the standard deviation of the time series and remain steady at this level. More details about the detection algorithm can be found in the Method Section. Hence, with this approach, the onset time of the postseismic surface displacement is controlled both by the noise level of the time series and the intensity of early postseismic displacement.

First of all, as shown by Fig. 3 and Table 1, ~43% of the time series exhibit a detectable surface displacement (i.e., 23 over 53 stations). This is a significant percentage considering that some of the stations are located far from the epicentres of the earthquakes (up to 250 km from the centroid). Then, for 18 of these 23 time series (i.e. ~78%), postseismic surface displacement can be detected within the first 2 hours after the earthquake, and several stations exhibit significant displacement at the surface as early as ten minutes after the earthquake. For stations with the most intense surface displacement (i.e., more than 30 mm measured after 12 hours), the average time before we can observe a significant signal at the surface is ~1 hour. When the surface displacement is less intense (i.e., between 20 and 30 mm measured after 12 hours), the time necessary to generate a detectable signal at the surface scatters over a large range (from 0 to 10 hours), and the noise level of the time series starts to play some role. Finally, no surface displacement can be reliably detected when the station does not show more than 20 mm of surface displacement after 12 hours.

To summarise, some stations show significant surface displacement as early as a few tens of minutes after the earthquake, and mainly within the first two hours. These observations suggest that the initiation of afterslip is very rapid, at least for the three earthquakes studied here. Thus, we observe a behaviour that is more similar to what is observed by Malservisi *et al*.^25^ than what is observed by Miyazaki and Larson^23^. Consequently, significant displacement might be missed when daily position time series are used, as they do not have the temporal resolution to capture the early displacement. This is what we attempt to quantify in the next section.

### Daily positioning is blind to a large fraction of the postseismic displacement

**Figure 4:**
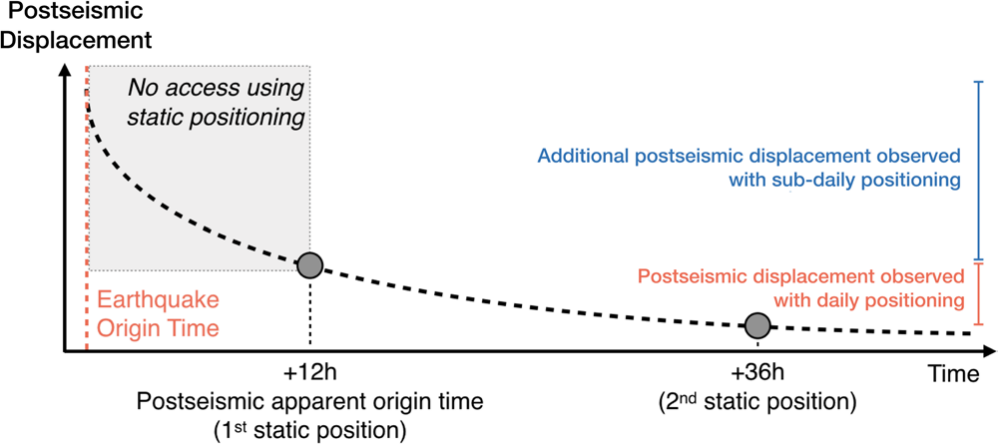
Schematic of the method used to assess the amount of postseismic displacement missed when daily positioning is used. The dashed line shows an idealised decaying postseismic trend. Standard postseismic observations start at the first daily solution, which can be seen as the postseismic apparent origin time. The displacement from the earthquake origin time up to the first daily position can only be resolved using sub-daily position time series.

When 24 hours of recorded data are reduced to a daily position, the effective time of the positioning is based on a weighted average of the available data. Assuming that the record does not contain gaps, the effective time of the position corresponds to the middle of the time window used to determine that position. As daily positioning strategy uses 24-hour blocks of data, the first point of the postseismic time series is on the day after the earthquake. Thus, with respect to the origin time of the earthquakes considered in this study – 06:34:12 UTC (Maule), 22:54:33 UTC (Illapel), and 23:58:37 UTC (Pedernales) – the effective time of the first daily position is 29.4 hours, 13.1 hours and 12.0 hours after the origin time. In some cases, if enough data are available between the earthquake origin time and the end of the day (23:59:59 UTC), a position can be obtained on the day of the earthquake. For instance, for the Maule earthquake, the data from 06:34:12 UTC to 23:59:59 UTC can be used to obtain a position on the day of the earthquake. In that case, the postseismic position time series will start ~9 hours after the earthquake. Consequently, the daily positioning strategy implies that a few hours of postseismic displacement are not included in the overall postseismic budget, despite being a time period when the slip-rate is supposed to be the largest (see Fig. 4). However, with high-rate kinematic position time series, we can quantify the amount of early postseismic displacement that is usually not included in daily position time series.

We use our high-rate position time series to mimic daily position time series. Because the time between the origin time of the earthquake and the first daily position varies from one earthquake to another, we assume a standard case in which the first daily position comes 12 hours after the mainshock. This is equivalent to a case where the earthquake occurs close to 23:59:59 UTC and it allows us to make conservative estimates with respect to the different possible scenarios. First, we select three windows that are 30 minutes long and centred around 30 minutes, 12 hours, and 36 hours after the earthquake origin time. For each window, we compute the average position. The difference between the position at 12 hours and that at 36 hours reproduce the traditional postseismic observation that can be made using daily positioning. On the other hand, the difference between the position at 30 minutes and that at 12 hours represent the amount of displacement that can only be observed using sub-daily positioning (see Fig. 4). We are aware that we only approximate the real case. Since daily positioning techniques use data over 24 hours, the obtained position do not actually stand on the kinematic position time series. Instead, it should be above (or below). It will tend towards the kinematic position time series as the rate of displacement becomes smaller and smaller. Thus, we slightly overestimate the difference compared to the real case.

**Figure 5:**
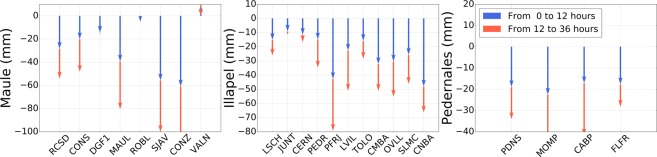
Comparison of the cumulative East displacement observed from 0 to 12 hours (blue arrows) and from 12 to 36 hours (red arrows). Note that the red arrows start at the tip of the blue arrows. Thus, the sum of the two arrows represents the cumulative East displacement over the first 36 hours.

Figure 5 summarises the results for all the stations for which significant postseismic displacement at the surface could be detected (see the previous Section). It shows that most of the displacement occurs within the first 12 hours. On average, for the East component, we find that ~64% of the displacement that is measured over 36 hours is in fact occurring during the first 12 hours. To the first order, this is consistent with a logarithmic decay of the postseismic displacement with a relaxation time of about 3.0 hours $(\frac{{\rm{l}}{\rm{o}}{\rm{g}}\,(1+12h/3h)}{{\rm{l}}{\rm{o}}{\rm{g}}\,(1+36h/3h)}\sim 63{\rm{ \% }})$. Thus, it clearly highlights the fact that a significant amount of surface displacement occurs very early after the earthquake, and that is not accounted for when daily positioning is used to study the postseismic phase.

## Discussion and Conclusive Remarks

In this study, first, we provide a detailed analysis on the emergence of postseismic surface displacement in high-rate kinematic position time series for three subduction zone earthquakes. This is a significant increase as it nearly doubles the number of observations on the early stage of the postseismic phase. These new observations will allow investigating the variability of behaviors in the early afterslip phase and how the transition from coseismic to postseismic occurs.

Second, our results indicate that the use of daily solutions to estimate the coseismic offsets introduces an average error of ~34%. That error depends on the amplitude of the offsets: (1) for offsets more than 50 cm, using daily positions leads to less than 10% of error, while (2) for offsets smaller than 50 cm, the error can be large (from ~0 to 200%). These estimates can have important implications for studies of the earthquake rupture, especially those using geodetic data (i.e., daily GNSS data and/or InSAR data). In particular, we can use them as guidelines to evaluate how much the coseismic slip distribution is contaminated by early afterslip. Indeed, in a purely elastic medium, as it is often assumed for inversions of coseismic slip, the early postseismic displacement included in the coseismic offsets will be mapped proportionately into the coseismic slip distribution. This is why studies that compare slip distributions inferred from seismic data only with those inferred from seismic and geodetic data find a discrepancy that is often around 10–30% *e*.*g*.^29,36^. We also want to point out that the processing routine used here is an efficient way to minimise the impact of early afterslip. It is preferable to daily solutions, which could be significantly contaminated by postseismic displacement, but it does not require the processing of very high-rate GNSS data (>1 Hz), which are not so often available.

Regarding the postseismic deformation itself, we observe that most of the examples available to us, meaning this study as well as those of Langbein *et al*.^9^, Munekane^26^ and Malservisi *et al*.^25^, show an almost immediate and intense start of the postseismic surface deformation. Thus, the observations for the Tokachi-Oki earthquake, which show an early postseismic signal that behaves in two phases^23^ appears to be an exception. Interestingly, this 2-phase behavior is predicted by the rate-and-state framework *e*.*g*.^20,24^, with the time scale of the acceleration phase (phase-I) ranging from 10^−6^ seconds up to 2 days^20^. Thus, it is possible that, for most cases, this initial acceleration phase is too short to be observed. It is also possible that, for most of the cases, the behavior of early postseismic deformation might simply be rate-dependent.

**Figure 6:**
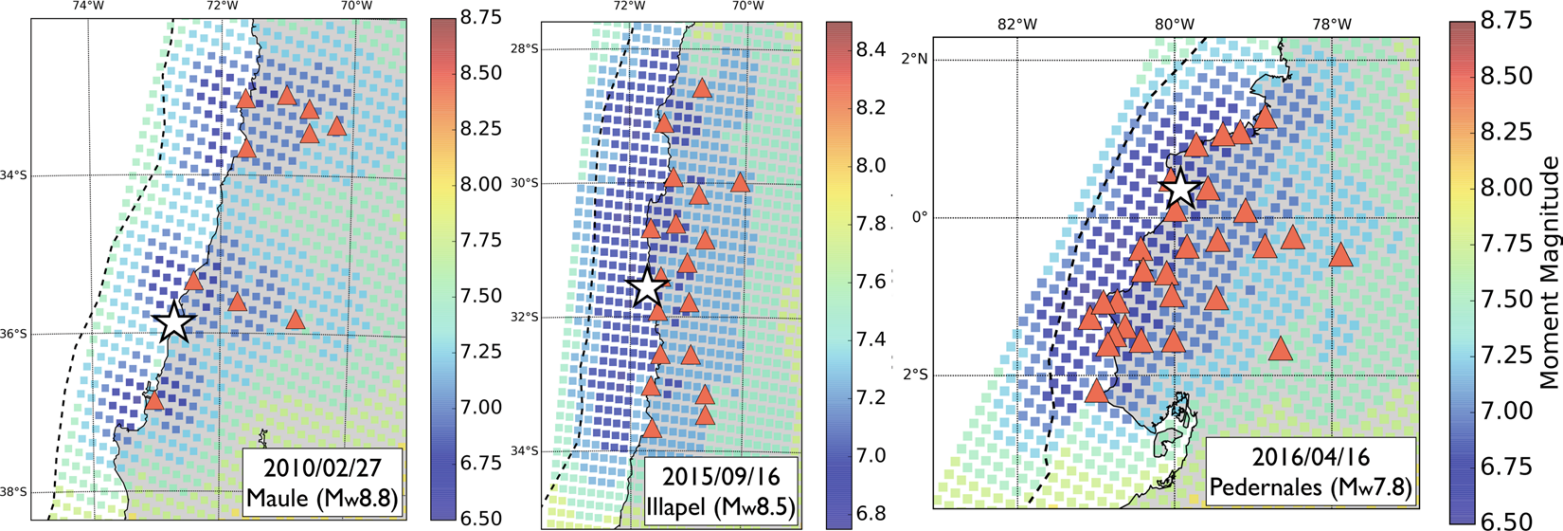
Moment magnitude of the afterslip that can be detected by the network based on the detection procedure used in this study (see the Method Section). The white star shows the location of the earthquake epicentre. The red triangles are the GNSS receivers. The continuous black line is the coast while the dashed black line shows the trench. The strike, dip and rake of the fault is that of the focal mechanism given by the GCMT catalog. The calculations are based on the approximation of a semi-infinite elastic half-space^59,60^.

An important question is to assess how much afterslip should be accumulated on the fault in order to generate a surface displacement that can be detected by our detection algorithm, the latter requiring a sustained surface displacement that is at least 3.3 times larger than the noise level. Since the average noise level over all of our time series is ~5 mm, it means that afterslip should generate more than 1.5 cm of surface displacement. To translate that in term of slip on the fault, we have performed a set of forward calculations (see Fig. 6). For each earthquake we create a distribution of dislocations consistent with the geometry of the slab. For each dislocation, we search for the equivalent moment magnitude (M_*eq*_) that generates more than 1.5 cm of surface displacement on the East component for at least one station of the network. The size of the dislocation depends on the moment magnitude and is determined using the scaling laws from Wells and Coppersmith^37^. We find that, for each earthquake, the network is able to detect afterslip if M_*eq*_ is larger than 6.5. Thus, we can estimate from our results that, for the three earthquakes, afterslip reaches an equivalent magnitude of 6.5 within the first two hours.

In any case, the fact that we can detect postseismic signal as early as a few tens of minutes after an earthquake advocate for the use of kinematic processing strategy to study the postseismic phase as early as possible after the mainshock. It suggests that we could get some early indications about the areas that are experiencing afterslip, which could have important implications regarding the assessment of areas of future large aftershocks. In their analysis of the 1992 Landers, California, earthquake (M_*w*_7.2), Perfettini and Avouac^38^ show that aftershocks and afterslip follow the same temporal evolution and are related in space to the stress changes induced by the progression of afterslip. More detailed studies are still needed, but it alludes to the possibility of using fast detection of early postseismic displacement to anticipate the areas that will host future aftershocks. This is even more critical since we know from the Omori’s law that the rate of aftershocks will decrease by 2 to 3 orders of magnitude after just one day (e.g., Enescu *et al*.^39^ on moderate-size earthquakes or Lengliné *et al*.^40^ on the 2011 Tohoku-Oki earthquake).

Regarding the intensity of the early postseismic phase, our results show that the cumulative displacement over the first 36 hours is essentially occurring during the first 12 hours, a time frame that is not fully accessible using daily positioning. Thus, it will affect the slip budget of afterslip. Over the 5 years after the Maule earthquake, Klein *et al*.^41^ have measured 40 and 70 cm of cumulative East displacement at stations CONZ and MAUL (Figs 1 and 11 of Klein *et al*.)^41^. Because they use daily position, the first point of their postseismic time series is on the day after the earthquake, i.e., about 30 hours of early postseismic displacement is missing. We have calculated that over this time period, ~9.6 cm and ~6.4 cm of displacement are measured on CONZ and MAUL, respectively, corresponding to about 25% and 10% of additional surface displacement. We reach a similar conclusion in the case of the 2016 Pedernales earthquake. Using daily positioning over a time period of 30 days, Rolandone *et al*.^42^ observe about 6.5 cm, 10.5 cm and 12.0 cm of cumulative East displacement for the stations PDNS, CABP and MOMP, respectively. In their study, the effective origin time of the postseismic time series is 12 hours after the earthquake. During the first 12 hours, we measure 1.7 cm, 2.2 cm and 1.7 cm of cumulative East displacement for the stations PDNS, CABP and MOMP, respectively, which corresponds to about 26%, 21% and 14% of additional surface displacement. These two examples show that the analysis of afterslip, when based on daily positioning only can strongly underestimate the amount of afterslip on the fault.

The fact that a significant amount of afterslip is missed can have major impacts on the overall postseismic slip budget. As it is used to assess if significant elastic stresses are still stored in the fault zone^43^, it can change the estimation of the seismic hazard on the fault and neighbouring faults. Also, since afterslip might represent barriers for large earthquakes^42^, understanding where early afterslip occurs will contribute to a more robust assessment of the seismogenic potential of a given fault. Finally, as a growing number of studies tend to link the occurrence of aftershocks and afterslip^44,45^, having information as early as possible about the intensity of afterslip could contribute to more reliable forecasting of potential large aftershocks.

To conclude, the current processing strategy of continuous high-rate GNSS data allows to better resolve the temporal evolution of afterslip, in particular at the time scale of the first few hours. We can now access the full surface displacement history at a given station from the first minutes after the earthquake, and up to several years. The accurate observation of the early postseismic stage is set to provide an enriched picture of the overall postseismic process and to shade light on the underlying physics.

## Methods

### Kinematic precise point positioning strategy

The high-rate position time series are obtained using the GD2P module of GIPSY-OASIS 6.4 software^46^ that is developed by the Jet Propulsion Laboratory (JPL). Our processing strategy is similar to that of Miyazaki and Larson^23^ and Malservisi *et al*.^25^. We use the precise point positioning strategy^47^ including the phase ambiguity resolution from a single receiver^48^. We use the final orbits and satellite clock estimates provided by JPL. We account for ocean loading effects using the FES2004 model^49^. The troposphere delays are calculated using the VMF1 mapping functions^50^. We account for higher order ionospheric terms using the IRI-2012b model^51^. We set the input parameters as suggested by the GIPSY-OASIS documentation except for two parameters. We use 9.0 × 10^−8^ km/$\sqrt{s}$ for the troposphere zenith random walk parameter as suggested by Selle and Desai^52^ and 3.0 × 10^−7^ km/$\sqrt{s}$ for the random walk parameter of the Kalman filter for the kinematic positioning according to Choi^53^.

We process 6 days before the earthquake, the day of the earthquake, as well as 3 days after the earthquakes. The six days before the earthquake are used to build the sidereal filter (see the next section). For each UTC day, we follow the flowchart described in Section A of the Supplementary Materials. First, the data are processed using a static strategy to estimate the troposphere delays and gradients (Step 1). These delays and gradients are used for the kinematic processing (Step 2). The obtained 30-seconds kinematic position time series is used for a new run with a static strategy, which refines the estimates of the troposphere delays and gradients (Step 3). Once again, the estimated delays and gradients are used to perform a new kinematic processing (Step 4). A final kinematic processing is performed (Step 5), which uses the obtained 30-seconds kinematic position time series from the previous Step.

As the maximum expected displacement from one epoch to another is directly dependent on the tuning of the random walk epoch-by-epoch position estimation, we prefer to remove the coseismic part in the observations, which produces larger dynamic displacement than the postseismic ones. Thus, for the day of the earthquake, the RINEX file is cut into two pieces. The pre-earthquake file stops 30 seconds before the earthquake origin time, and the post-earthquake file starts 5 minutes after the earthquake origin time. The 5-minutes threshold was defined so that a Rayleigh wave leaving the centroid after 200 seconds (i.e., the source duration of the Maule earthquake) and at a speed of 2.7 km/s (i.e., 90% of the shear wave speed) has passed the farthest station of each network. Each step described above is performed for each piece independently, except for Step 3. For this Step, we estimate the troposphere delays and gradients using the full RINEX file, and using the two kinematic position time series from Step 2, merged together. This is to avoid any discontinuity in the troposphere parameter estimation.

To minimise the discontinuities at the UTC day transition, we process 30-hours long RINEX file (i.e., from 21:00:00 UTC of day minus one to 03:00:00 UTC of day plus one). Thus, each position time series overlap with the next one over a six-hour time window. We merge successive time series by choosing the point within the overlapping time window when the difference between the two time series is minimum.

The quality of the strategy is quantified using the reduction of the post-fit residuals for the LC phase combination for each step of the processing. The average LC residuals for the static runs (Step 1 and 3) are 9.9 × 10^−6^ km and 8.8 × 10^−6^ km. Thus, using apriori position time series rather than a constant apriori position leads to a 10% reduction of the phase residuals. For the kinematic runs (Step 2, 4 and 5), we get 9.1 × 10^−6^ km, 9.0 × 10^−6^ km, and 8.8 × 10^−6^ km, meaning that the multi-step strategy leads to an overall 3% reduction of the phase residuals.

### Sidereal filtering

First, we use the 6 days before the earthquake to estimate a linear trend that is removed from the entire time series. Then, we attempt to minimise the effects of multi-paths and other kinds of perturbations caused by the geometry of the satellites by applying a sidereal filter to the time series^36,54^.

Choi *et al*.^36^ have shown that the sidereal period is not the same for all satellites. Thus, they suggest using the same set of satellites over the entire time period that is processed to ensure that the estimated sidereal period is appropriate at all times. This approach has the disadvantage of reducing the number of satellites used to obtain the position time series. In addition, the traditional sidereal filter relies on the use of a ~24 h window to filter the next ~24 h window, and so on. Thus, if the time series exhibits a significant trend over a long time, as we might expect for the postseismic deformation, the filtering might introduce spurious effects. To overcome this issue, we adopt a different approach and use a sidereal filter that is based on cross-correlating successive days *e*.*g*.^55^. The idea is to constructively stack the repeating patterns over several days, only on days when no earthquake or postseismic trend is observed, i.e., on the 6 days before the earthquake. Because the sidereal filter is built over 6 days, we believe that it is representative of the sidereal signature of the site over the whole time period that we want to filter, eliminating the need of constant satellite constellation through time. In addition, we can filter the postseismic time period without introducing artifacts that might arise because of the significant postseismic trend, which we do not want to remove.

**Figure 7:**
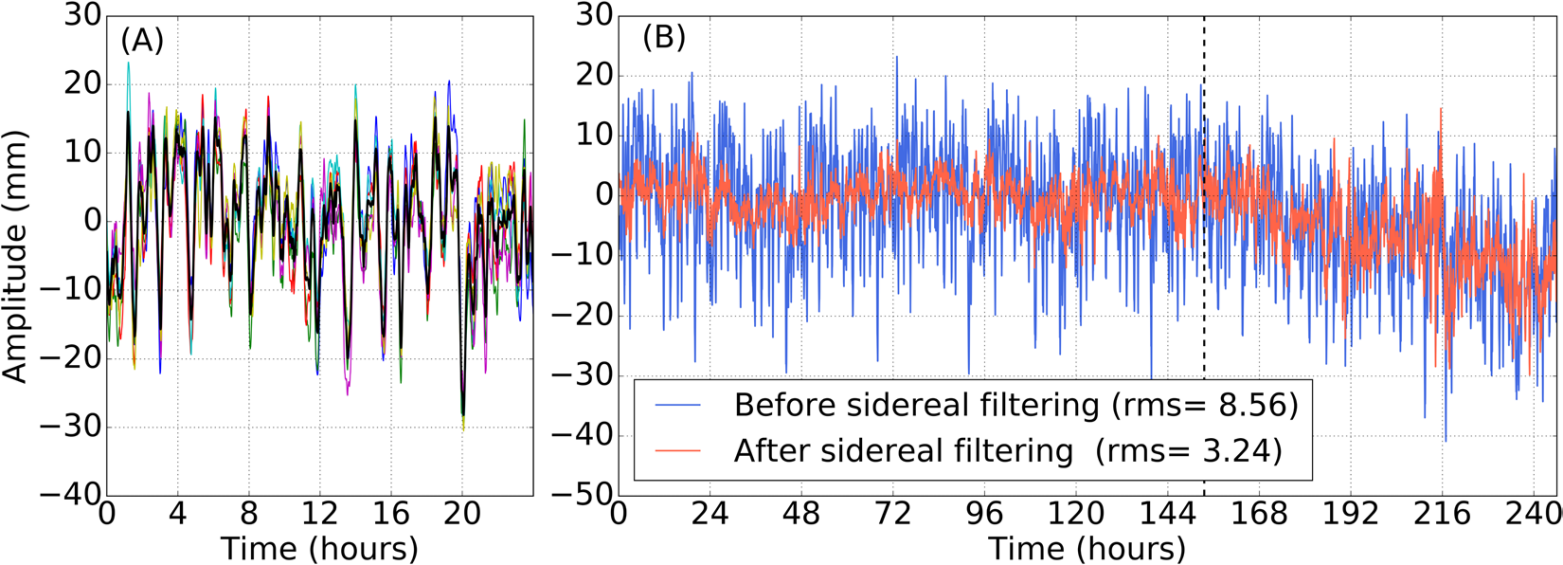
East position time series for station VNEV around the time of the 2010 Maule earthquake (see Fig. 1). (**A**) Sidereal filter. The coloured lines represent the six 24-hour time series preceding the mainshock. The black line is the average of the 6 time series after that they have been properly shifted and stacked. This is the sidereal filter that is going to be removed from the time series. (**B**) Position time series before applying the sidereal filter (blue) and after applying the sidereal filter (red). For this specific case, the standard deviation (or RMS) of the time series, calculated using the data before the mainshock, has been reduced from 8.6 mm to 3.5 mm. The vertical dashed line shows the time of the earthquake. Note that the coseismic offset has been removed.

In practice, we cross-correlate the first two days to determine the time lag that maximises the cross-correlation. Then, we shift and stack these two days to produce the first version of the sidereal filter. After that, we cross-correlate the sidereal filter with the third day. Again, we shift and stack the third day with the sidereal filter based on the cross-correlation. This process is repeated for the 6 days that precede the mainshock. Once built, we remove the mean and the linear trend of the sidereal filter. Then, we cross-correlate the sidereal filter with each day of the entire time series and the sidereal filter is then removed from the time series (see Fig. 7). To ensure that we are not introducing spurious effects, we only apply the filter if, during its construction, the average cross-correlation between the different days and the sidereal filter is above 0.3. The different figures in Section B of the Supplementary Materials summarise the effectiveness of the filter by showing the reduction of the standard deviation of the time series after applying the sidereal filter. It shows that the standard deviation of the time series goes on average from 6.8 mm to 4.8 mm on the North component, from 7.1 to 5.0 mm on the East component and from 13.5 to 10.0 mm on the Vertical component.

### Detecting the onset time of postseismic displacement

To detect the onset time of the postseismic displacement, we design an algorithm to estimate when the mean of the position time series changes significantly and remains at its new level. For that, we assume that the position time series follow a normal distribution. The algorithm for the detection is based on the Chow-test^35^, which tests the significance of using two linear regressions to model a given dataset.

The null hypothesis assumes that the time series do not exhibit a change of mean. Thus, the residual sum of squares for the null hypothesis is:

1 $${S}_{0}=\sum _{i=1}^{N}{({u}_{i}-\bar{u})}^{2}$$

where *u*_*i*_ is the position at time *i*, and $\bar{u}$ is the mean of the time series over N points. The alternative hypothesis is that there is a change of mean of the time series at a given break point *τ*. Similarly, we can compute the residual sum of squares for the two sets:

2 $${S}_{12}={S}_{1}+{S}_{2}=\sum _{i=1}^{\tau }{({u}_{i}-\bar{u})}^{2}+\sum _{j=\tau +1}^{N}{({u}_{j}-\bar{u})}^{2}$$

Once we have computed these two quantities, we can compute the Chow-test statistic that is:

3 $$\chi =\frac{({S}_{0}-{S}_{12})/k}{{S}_{12}/({N}_{1}+{N}_{2}-2k)}$$

where *N*_1_ and *N*_2_ are the number of observations in each group and *k* is the number of parameters (in this case *k* = 1).

In practice, we slide a 12-hour long window (i.e., 1440 points) over the entire time series with a step of 30 seconds. At each step, we compute *χ* assuming that the potential break point is at the centre of the window (i.e., *τ* = 720 points and *N*_1_ = *N*_2_ = 720 points). Like this, we test every point as a potential break point. Then, several criteria are applied to determine the point when we start to observe significant postseismic displacement.

**Figure 8:**
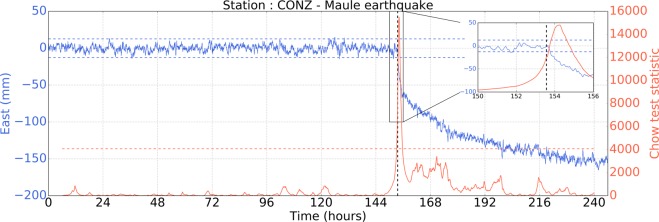
The blue curve shows the East position time series at station CONZ around the time of the 2010 Maule earthquake (see Fig. 1). Note that the coseismic offset has been removed from the time series and that the gap corresponding to the passing of seismic waves has been filled with zeros. The blue horizontal dashed lines show the noise level (i.e., the mean of the time series before the earthquake plus and minus 3.3 times the standard deviation of the time series, also calculated from the time series before the earthquake). The vertical dashed line is the earthquake origin time. The red line is the time evolution of the Chow-test statistic (*χ*) The red horizontal dashed line is the threshold to identify potential ties when significant postseismic deformation might occur. Finally, the detection time is validated if the peak is located after the earthquake origin time and is more than 70% of the time series after the peak remains outside the noise level. For instance, on this figure, significant postseismic deformation is detected ~41 minutes after the earthquake.

First, we identify all the peaks in the *χ* time series that are above 3.3 times its own standard deviation, giving us a set of potential time for the break point. Then, we only consider those that are after the earthquake origin time. Finally, we go through all of them and, for each, we test whether at least 70% of the time series after the peak has a mean that differs from the pre-seismic mean by at least 3.3 times the RMS of the time series. We set the onset time of the postseismic displacement to be the first one in time that successfully pass all the criteria. Figure 8 illustrates this method for a given time series.

Since various friction laws can generate various types of surface deformation, we do not know what type of signal to expect and prefer not to filter the time series. A low-pass filter would remove some noise but also potentially cause artifacts. The time when the signal rises above the noise level can thus be seen as an upper bound.

## Supplementary information

Supplementary Files
