# Supplementary material for: Unravelling the contribution of early postseismic deformation using sub-daily GNSS positioning
Source: Sci Rep. 2019 Feb 11;9:1775. doi: 10.1038/s41598-019-39038-z (PMC6370855; doi:10.1038/s41598-019-39038-z)
Supplement: Supplementary file 1 — Supplementary Files [file 41598_2019_39038_MOESM1_ESM.pdf]

Unravelling the contribution of early postseismic deformation using sub-daily GNSS positioning

Cedric Twardzik[1,*], **Mathilde Vergnolle**[1], **Anthony Sladen**[1], **and Antonio Avallone**[2]

[1]Université Côte d'Azur, CNRS, Observatoire de la Côte d'Azur, IRD, Geoazur, UMR 7329, 250 rue Albert Einstein, Sophia-Antipolis, 06560, Valbone, France

[2]Istituto Nazionale di Geofisica e Vulcanologia, Osservatorio Nazionale Terremoti, Via di Vigna Murata 605, Rome, 00143, Italy

[*]cedric.twardzik@geoazur.unice.fr

SUPPLEMENTARY MATERIALS

Supplementary Materials

A Kinematic precise point positioning strategy

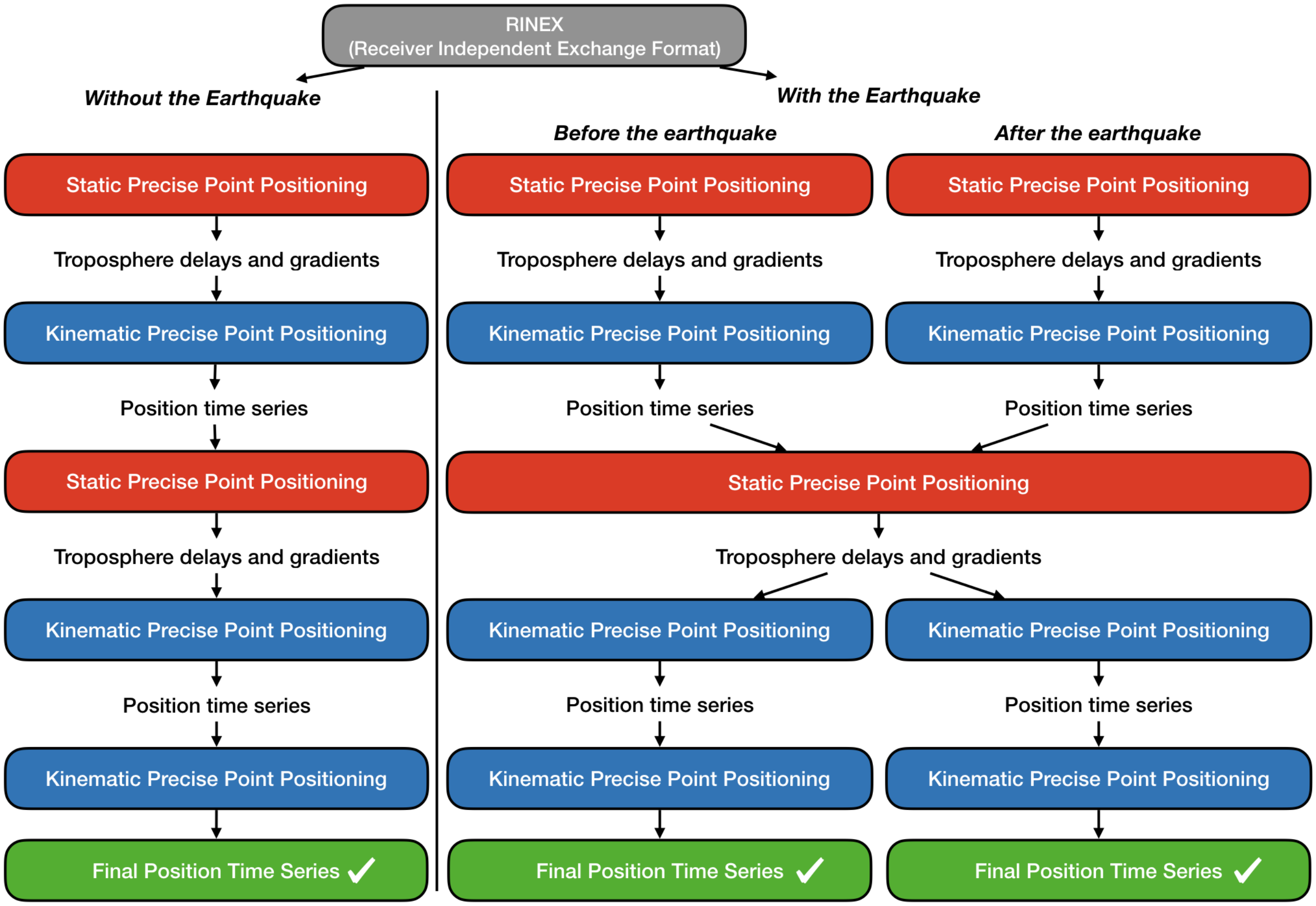

Figure A.1. Schematic that illustrates the processing strategy of the GNSS data. The strategy is mostly based on[25]. Note that the processing is different if the RINEX file contains the earthquake. Regarding the different parameters of the processing: (1) the static runs are done at a sampling rate of 300 seconds while the kinematic runs are done at a sampling rate of 30 seconds. (2) We use a satellite cutoff angle of 7°. (3) Each satellite must be locked for at least 20 minutes to be considered during the processing. (4) All other parameters are set up as recommended by the JPL documentation. Figure A.2 shows a pseudo-code of the processing routine.

```
> teqc -O.obs L1L2C1C2P1P2 -O.s G -R -O.st YYYY mm (DD-1) 21 00 00.00 -O.e YYYY mm (DD+1) 03 00 00.00 RINEX.LIST > RINEX.NEW
> clockprep -i RINEX.NEW -o RINEX.PREP -fixonlyphase -nocopy
> tropnominal -n XXXX -m VMF1GRID -latdeg 00.00 -londeg 00.00 -h_m 00.00 -stsec 00.00 -endsec 00.00 -samp 300
> cat XXXX.TDPdry XXXX.TDPwet > XXXX.TDPdryandwet
> antex2xyz -antexfile igs14_1958.atx -xyzfile XXXX_antex.xyz -anttype XX -recname XXXX -radcode XX -fel 0 -del 5 -daz 5 -extrap

> (gd2p.pl -i RINEX.PREP -n XXXX -d YYYY-mm-DD -r 300 -type s -w_elmin 7 -eldepwght SQRTSIN
    -e "-a 20 -PC -LC -F -t1 TSTART -t2 TSTOP" -pb_min_slip 1.0E-3 -pb_min_elev 30 -amb_res 2 -dwght 1.0E-5 1.0E-3
    -post_wind 5.0E-3 5.0E-5 -trop_z_rw 9.0E-8 -wetzgrad 5.0E-9 -trop_map VMF1GRID -tdp_in XXXX.TDPdryandwet
    -tides WahrK1 FreqDepLove OctTid PolTid -add_ocnld " -c FES2004.COEFF" -OcnldCpn -add_ocnldpoltid  -ion_2nd
    -shell_height 600 -tec_mdl iri -orb_clk "flinnR /JPLORBCLK" -AntCal XXXX_antex.xyz
    -p 1234.56789 1234.56789 1234.56789 -env_km 0.0 0.0 0.00001234 -stacov > gd2p.log ) >& gd2p.err

!! In case of an earthquake, run two times: One time up to 30s before the earthquake and one time 150s after the earthquake !!

> grep -P 'TRP|WETZ' tdp_final > XXXX_STATIC.TDPwetanddry ; cat XXXX.TDPdry >> XXXX_STATIC.TDPwetanddry


> (gd2p.pl -i RINEX.PREP -n XXXX -d YYYY-mm-DD -r 30 -type k -w_elmin 7 -eldepwght SQRTSIN
    -e "-a 20 -PC -LC -F -t1 TSTART -t2 TSTOP" -pb_min_slip 1.0E-3 -pb_min_elev 30 -amb_res 2 -dwght 1.0E-5 1.0E-3
    -post_wind 5.0E-3 5.0E-5 -trop_z_rw 9.0E-8 -wetzgrad 5.0E-9 -trop_map VMF1GRID -tdp_in XXXX_STATIC.TDPwetanddry
    -tides WahrK1 FreqDepLove OctTid PolTid -add_ocnld " -c FES2004.COEFF" -OcnldCpn -add_ocnldpoltid -ion_2nd
    -shell_height 600 -tec_mdl iri -orb_clk "flinnR /JPLORBCLK" -AntCal XXXX_antex.xyz
    -p 1234.56789 1234.56789 1234.56789 -env_km 0.0 0.0 0.00001234 -stacov
    -kin_sta_xyz 1.0E-3 3.0E-7 30 RANDOMWALK > gd2p.log ) >& gd2p.err

!! In case of an earthquake, run two times: One time up to 30s before the earthquake and one time 150s after the earthquake !!

!! if no earthquake:
    > grep 'STA [XYZ]' tdp_final > XXXX_KINE.TDPstawetanddry ; cat XXXX.TDPdryandwet >> XXXX_KINE.TDPstawetanddry
!! if earthquake:
    > grep 'STA [XYZ]' tdp_final_before >     XXXX_KINE.TDPstawetanddry
    > grep 'STA [XYZ]' tdp_final_after    >> XXXX_KINE.TDPstawetanddry
    > cat XXXX.TDPdryandwet               >> XXXX_KINE.TDPstawetanddry

> (gd2p.pl -i RINEX.PREP -n XXXX -d YYYY-mm-DD -r 300 -type s -w_elmin 7 -eldepwght SQRTSIN
    -e "-a 20 -PC -LC -F -t1 TSTART -t2 TSTOP" -pb_min_slip 1.0E-3 -pb_min_elev 30 -amb_res 2 -dwght 1.0E-5 1.0E-3
    -post_wind 5.0E-3 5.0E-5 -trop_z_rw 9.0E-8 -wetzgrad 5.0E-9 -trop_map VMF1GRID -tdp_in XXXX_KINE.TDPstawetandry
    -tides WahrK1 FreqDepLove OctTid PolTid -add_ocnld " -c FES2004.COEFF" -OcnldCpn -add_ocnldpoltid -ion_2nd
    -shell_height 600 -tec_mdl iri -orb_clk "flinnR /JPLORBCLK" -AntCal XXXX_antex.xyz
    -p 1234.56789 1234.56789 1234.56789 -env_km 0.0 0.0 0.00001234 -stacov > gd2p.log ) >& gd2p.err

> grep -P 'TRP|WETZ' tdp_final > XXXX_STATIC.TDPwetanddry ; cat XXXX.TDPdry >> XXXX_STATIC.TDPwetanddry

> (gd2p.pl -i RINEX.PREP -n XXXX -d YYYY-mm-DD -r 30 -type k -w_elmin 7 -eldepwght SQRTSIN
    -e "-a 20 -PC -LC -F -t1 TSTART -t2 TSTOP" -pb_min_slip 1.0E-3 -pb_min_elev 30 -amb_res 2 -dwght 1.0E-5 1.0E-3
    -post_wind 5.0E-3 5.0E-5 -trop_z_rw 9.0E-8 -wetzgrad 5.0E-9 -trop_map VMF1GRID -tdp_in XXXX_STATIC.TDPwetanddry
    -tides WahrK1 FreqDepLove OctTid PolTid -add_ocnld " -c FES2004.COEFF" -OcnldCpn -add_ocnldpoltid
    -ion_2nd -shell_height 600 -tec_mdl iri -orb_clk "flinnR /JPLORBCLK" -AntCal XXXX_antex.xyz
    -p 1234.56789 1234.56789 1234.56789 -env_km 0.0 0.0 0.00001234 -stacov
    -kin_sta_xyz 1.0E-3 3.0E-7 30 RANDOMWALK > gd2p.log ) >& gd2p.err

!! In case of an earthquake, run two times: One time up to 30s before the earthquake and one time 150s after the earthquake !!

> cp XXXX_STATIC.TDPdryandwet XXXX_KINE_STATIC.TDPstawetanddry
> grep 'STA [XYZ]' tdp_final >> XXXX_KINE_STATIC.TDPstawetanddry

> (gd2p.pl -i RINEX.PREP -n XXXX -d YYYY-mm-DD -r 30 -type k -w_elmin 7 -eldepwght SQRTSIN
    -e "-a 20 -PC -LC -F -t1 TSTART -t2 TSTOP" -pb_min_slip 1.0E-3 -pb_min_elev 30 -amb_res 2 -dwght 1.0E-5 1.0E-3
    -post_wind 5.0E-3 5.0E-5 -trop_z_rw 9.0E-8 -wetzgrad 5.0E-9 -trop_map VMF1GRID -tdp_in XXXX_KINE_STATIC.TDPstawetanddry
    -tides WahrK1 FreqDepLove OctTid PolTid -add_ocnld " -c FES2004.COEFF" -OcnldCpn -add_ocnldpoltid
    -ion_2nd -shell_height 600 -tec_mdl iri -orb_clk "flinnR /JPLORBCLK" -AntCal XXXX_antex.xyz
    -p 1234.56789 1234.56789 1234.56789 -env_km 0.0 0.0 0.00001234 -stacov
    -kin_sta_xyz 1.0E-3 3.0E-7 30 RANDOMWALK > gd2p.log ) >& gd2p.err

!! In case of an earthquake, run two times: One time up to 30s before the earthquake and one time 150s after the earthquake !!
```

Figure A.2. Pseudo-code of the processing routine

B Effectiveness of the sidereal filter

The next three figures show the RMS of the time series before and after applying the sidereal filter on all stations in this study. The RMS is the standard deviation of the time series using the positions prior to the earthquake origin time. We show that the effect of the sidereal filter for all stations but we only apply it when the cross-correlation measured during the construction of the filter is greater than 0.3 (see the Method Section). Stations for which the sidereal filter is applied are highlighted by a red square.

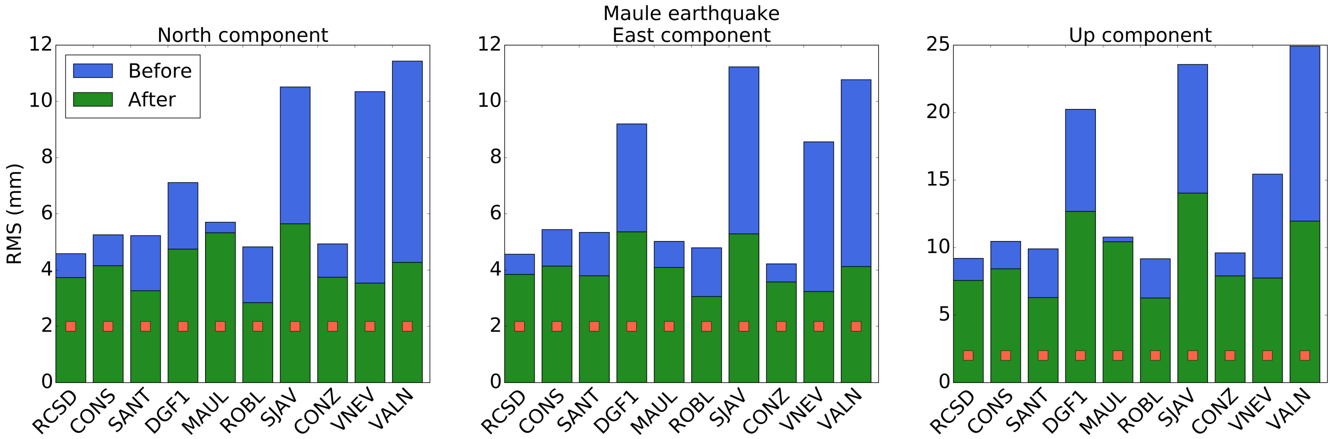

Figure B.1. Quantification of the noise reduction after applying the sidereal filter on each component of each station for the 2010 Maule, Chile, earthquake. The blue bars show the noise level before applying the sidereal filter and the green bars after applying the sidereal filter. The red squares highlight the stations for which we apply the sidereal filter. On average, the RMS is reduced by 38% on the North component, 34% on the East component, and 31% on the Up component.

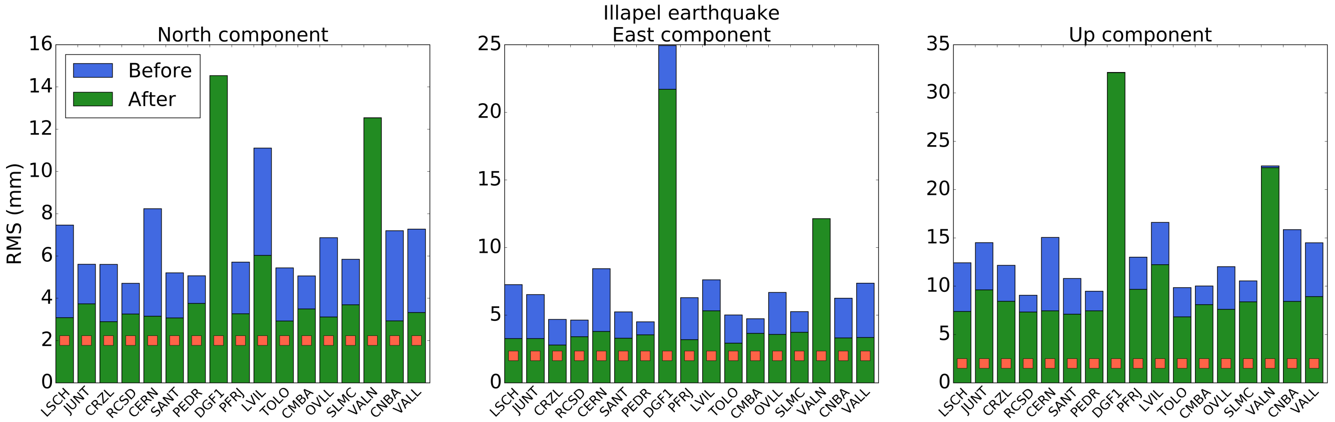

Figure B.2. Quantification of the noise reduction after applying the sidereal filter on each component of each station for the 2015 Illapel, Chile, earthquake. The blue bars show the noise level before applying the sidereal filter and the green bars after applying the sidereal filter. The red squares highlight the stations for which we apply the sidereal filter. On average, the RMS is reduced by 40% on the North component, 32% on the East component, and 27% on the Up component.

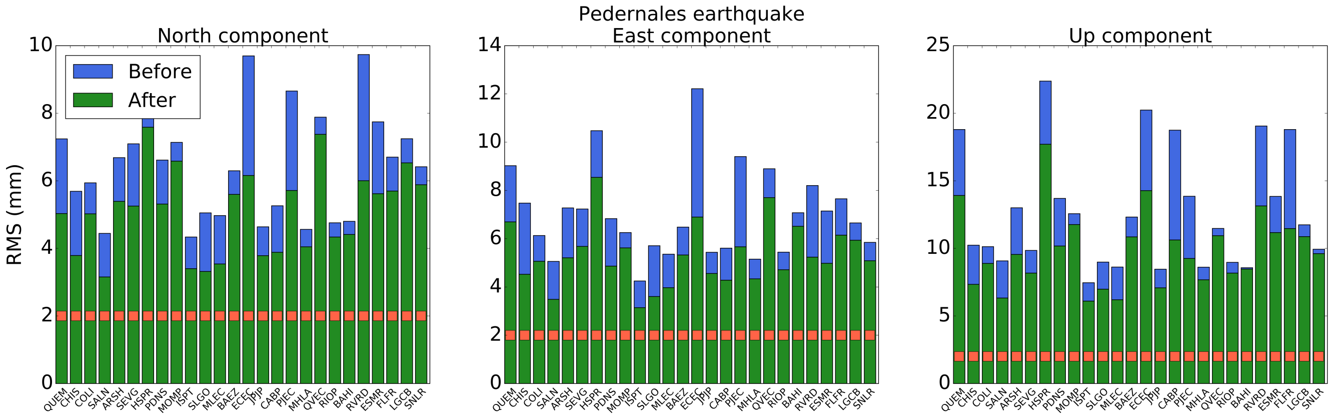

Figure B.3. Quantification of the noise reduction after applying the sidereal filter on each component of each station for the 2016 Pedernales, Ecuador, earthquake. The blue bars show the noise level before applying the sidereal filter and the green bars after applying the sidereal filter. The red squares highlight the stations for which we apply the sidereal filter. On average, the RMS is reduced by 22% on the North component, 10% on the East component, and 18% on the Up component.

C Error on the coseismic offsets

The table below summarises the data used to produce Figure 2 in the main text. Note that the calculations are made only for the East component. In brackets, we show the percentage of difference with respect to the strict coseismic offsets. The term 1 day, 2 days and 3 days are the different time windows that we use to compute the average position on either side of the earthquake origin time.

Maule earthquake:				
Station name	strict offsets (mm)	1 day offsets (mm)	2 days offsets (mm)	3 days offsets (mm)
RCSD	-676.4003	-713.4804 (5.48)	-723.8694 (7.02)	-730.0030 (7.92)
CONS	-4663.2833	-4697.7939 (0.74)	-4707.8679 (0.96)	-4715.1020 (1.11)
SANT	-248.5500	-248.0187 (0.21)	-250.7926 (0.90)	-251.9147 (1.35)
DGF1	-413.0500	-428.5607 (3.76)	-433.4208 (4.93)	-435.6112 (5.46)
MAUL	-996.3750	-1048.9133 (5.27)	-1062.3350 (6.62)	-1072.2166 (7.61)
ROBL	-154.6400	-166.5790 (7.72)	-169.2466 (9.45)	-170.0421 (9.96)
SJAV	-2279.6000	-2338.8814 (2.60)	-2356.7265 (3.38)	-2368.3783 (3.89)
CONZ	-2817.4000	-2903.8465 (3.07)	-2922.7570 (3.74)	-2934.7930 (4.17)
VNEV	-295.6667	-296.9626 (0.44)	-298.7592 (1.05)	-300.8467 (1.75)
VALN	-87.2333	-101.6065 (16.48)	-103.2041 (18.31)	-104.6905 (20.01)

Table C.1. Details of the values used to produce Figure 2 in the main text.

Illapel earthquake:				
Station name	strict offsets (mm)	1 day offsets (mm)	2 days offsets (mm)	3 days offsets (mm)
LSCH	-156.8571	-171.0205 (9.03)	-174.7044 (11.38)	-177.1400 (12.93)
JUNT	-113.1571	-128.5675 (13.62)	-128.7922 (13.82)	-130.0923 (14.97)
CRZL	-23.1000	-22.8193 (1.22)	-23.9411 (3.64)	-23.5998 (2.16)
RCSD	-2.4143	-1.3604 (43.65)	-1.2431 (48.51)	-0.1024 (95.76)
CERN	-47.4571	-61.6560 (29.92)	-63.7426 (34.32)	-65.0911 (37.16)
SANT	-18.0143	-21.3808 (18.69)	-22.5605 (25.24)	-22.9200 (27.23)
PEDR	-510.7000	-538.7434 (5.49)	-544.9800 (6.71)	-548.5015 (7.40)
DGF1	-14.1667	5.6892 (140.16)	-2.9523 (79.16)	-1.5298 (89.20)
PFRJ	-1380.5571	-1439.1194 (4.24)	-1452.3871 (5.20)	-1460.9975 (5.83)
LVIL	-285.0857	-355.5782 (24.73)	-366.5318 (28.57)	-372.9432 (30.82)
TOLO	-226.9143	-253.8072 (11.85)	-258.5451 (13.94)	-261.2538 (15.13)
CMBA	-809.8991	-862.3400 (6.47)	-869.6235 (7.37)	-873.7081 (7.88)
OVLL	-693.3714	-735.9753 (6.14)	-743.7877 (7.27)	-748.6480 (7.97)
SLMC	-352.9571	-397.6396 (12.66)	-405.1986 (14.80)	-408.9895 (15.88)
VALN	106.9017	2.4730 (97.69)	-1.5320 (101.43)	-3.5481 (103.32)
CNBA	-1187.5429	-1251.7268 (5.40)	-1260.4030 (6.14)	-1264.1526 (6.45)
VALL	-4.5333	-14.5864 (221.76)	-14.5344 (220.61)	-13.4591 (196.89)

Table C.2. Details of the values used to produce Figure 2 in the main text.

Pedernales earthquake:				
Station name	strict offsets (mm)	1 day offsets (mm)	2 days offsets (mm)	3 days offsets (mm)
QUEM	-59.2500	-51.2707 (13.47)	-54.1553 (8.60)	-53.7952 (9.21)
CHIS	-27.2286	-15.8598 (41.75)	-15.3256 (43.72)	-16.7118 (38.62)
COLI	7.5001	-9.1112 (221.48)	-8.5194 (213.59)	-8.0671 (207.56)
SALN	-23.6571	-11.7961 (50.14)	-10.3532 (56.24)	-9.7174 (58.92)
ARSH	-105.4000	-108.7341 (3.16)	-109.9424 (4.31)	-110.7700 (5.09)
SEVG	-48.0857	-34.1320 (29.02)	-32.6949 (32.01)	-32.3760 (32.67)
HSPR	-99.6286	-83.7463 (15.94)	-81.5415 (18.15)	-79.8990 (19.80)
PDNS	-666.5013	-682.9228 (2.46)	-689.6675 (3.48)	-693.7728 (4.09)
MOMP	-84.8143	-106.2321 (25.25)	-114.6005 (35.12)	-120.0085 (41.50)
ISPT	-6.5857	-2.8066 (57.38)	-1.1038 (83.24)	-2.0310 (69.16)
SLGO	8.3429	-6.7067 (180.39)	-5.4153 (164.91)	-4.7036 (156.38)
MLEC	-59.4571	-60.1958 (1.24)	-59.4273 (0.05)	-60.8047 (2.27)
BAEZ	-30.1143	-24.8603 (17.45)	-23.6291 (21.54)	-22.9700 (23.72)
ECEC	-246.5429	-206.0553 (16.42)	-201.8984 (18.11)	-202.7029 (17.78)
JPJP	-23.3143	-8.5671 (63.25)	-7.6650 (67.12)	-7.7974 (66.56)
CABP	-525.1429	-543.3145 (3.46)	-550.6044 (4.85)	-556.3711 (5.95)
PJEC	-13.5286	-5.1175 (62.17)	-4.4238 (67.30)	-3.5603 (73.68)
MHLA	-4.6286	-7.9820 (72.45)	-6.0295 (30.27)	-5.5969 (20.92)
QVEC	-51.2993	-54.4295 (6.10)	-54.3216 (5.89)	-53.9211 (5.11)
BAHI	-123.7003	-123.1828 (0.42)	-124.0904 (0.32)	-124.5988 (0.73)
RVRD	-8.4000	-20.4554 (143.52)	-18.3902 (118.93)	-16.7223 (99.08)
ESMR	-25.3005	-23.6292 (6.61)	-24.0841 (4.81)	-23.7315 (6.20)
FLFR	-356.2286	-394.9591 (10.87)	-397.5719 (11.61)	-399.1921 (12.06)
LGCB	-185.0993	-189.8746 (2.58)	-193.1560 (4.35)	-194.2354 (4.94)
SNLR	-8.4000	-13.4473 (60.09)	-12.3391 (46.89)	-12.2502 (45.84)

Table C.3. Details of the values used to produce Figure 2 in the main text.

D Detection of the onset time

Earthquake name	Station name	Detection Time (hours)	Amplitude at 12 hours (mm)	Noise level (mm)	Distance to the centroid (km)
MAULE	RCSD	1.280	-36.381	4.557	294.174
MAULE	CONS	1.313	-33.830	4.141	98.246
MAULE	SANT	-1.000	2.008	3.798	388.078
MAULE	DGF1	1.547	-18.096	5.358	361.090
MAULE	MAUL	0.713	-53.778	4.096	210.600
MAULE	ROBL	0.072	-12.805	3.061	387.060
MAULE	SJAV	1.522	-65.386	5.291	134.818
MAULE	CONZ	0.680	-88.582	4.223	96.690
MAULE	VNEV	-1.000	0.615	3.235	394.515
MAULE	VALN	6.255	-8.791	4.126	356.389

Table D.1. Details about the data used to produce Figure 3 and Table 1 in the main text. Note that the detection procedure is only applied on the East component. Note that when the detection time is -1, it means that no significant postseismic signal has been detected.

Earthquake name	Station name	Detection Time (hours)	Amplitude at 12 hours (mm)	Noise level (mm)	Distance to the centroid (km)
ILLAPEL	LSCH	3.283	-15.504	3.277	158.091
ILLAPEL	JUNT	3.383	-15.077	3.275	230.198
ILLAPEL	CRZL	-1.000	0.044	2.801	234.841
ILLAPEL	RCSD	-1.000	1.002	3.411	284.244
ILLAPEL	CERN	0.074	-14.883	3.806	192.986
ILLAPEL	SANT	-1.000	-2.261	3.302	261.483
ILLAPEL	PEDR	5.483	-27.163	3.559	137.406
ILLAPEL	DGF1	-1.000	9.302	24.296	291.523
ILLAPEL	PFRJ	0.249	-62.375	3.197	66.659
ILLAPEL	LVIL	0.408	-72.341	5.332	102.422
ILLAPEL	TOLO	0.066	-29.404	2.944	162.734
ILLAPEL	CMBA	0.391	-54.267	3.667	104.016
ILLAPEL	OVLL	0.591	-43.068	3.584	102.844
ILLAPEL	SLMC	0.433	-44.927	3.745	128.873
ILLAPEL	VALN	-1.000	-134.281	11.615	215.349
ILLAPEL	CNBA	0.416	-67.826	3.324	67.069
ILLAPEL	VALL	-1.000	-7.456	3.358	311.841

Table D.2. Details about the data used to produce Figure 3 and Table 1 in the main text. Note that the detection procedure is only applied on the East component. Note that when the detection time is -1, it means that no significant postseismic signal has been detected.

Earthquake name	Station name	Detection Time (hours)	Amplitude at 12 hours (mm)	Noise level (mm)	Distance to the centroid (km)
PEDERNALES	QUEM	-1.000	8.423	6.695	195.319
PEDERNALES	CHIS	-1.000	7.447	4.524	116.593
PEDERNALES	COLI	-1.000	-17.876	5.065	161.012
PEDERNALES	SALN	-1.000	10.545	3.486	244.066
PEDERNALES	ARSH	-1.000	-5.192	5.207	130.314
PEDERNALES	SEVG	-1.000	13.998	5.680	97.931
PEDERNALES	HSPR	-1.000	23.306	8.545	157.762
PEDERNALES	PDNS	5.873	-17.594	4.864	38.621
PEDERNALES	MOMP	1.315	-27.777	6.255	71.725
PEDERNALES	ISPT	-1.000	1.466	3.141	156.568
PEDERNALES	SLGO	-1.000	-16.213	3.606	177.539
PEDERNALES	MLEC	-1.000	-0.205	3.969	128.016
PEDERNALES	BAEZ	-1.000	5.920	6.455	265.492
PEDERNALES	ECEC	-1.000	49.985	6.898	90.339
PEDERNALES	JPJP	-1.000	11.835	5.436	145.481
PEDERNALES	CABP	0.256	-20.113	4.279	35.676
PEDERNALES	PJEC	-1.000	5.142	5.665	160.462
PEDERNALES	MHLA	-1.000	-6.337	4.339	161.709
PEDERNALES	QVEC	-1.000	-5.908	8.879	131.808
PEDERNALES	RIOP	-1.000	10.549	5.440	246.101
PEDERNALES	BAHI	-1.000	0.169	7.070	62.140
PEDERNALES	RVRD	-1.000	-12.804	5.238	163.365
PEDERNALES	ESMR	-1.000	4.118	4.981	131.028
PEDERNALES	FLFR	0.106	-41.018	6.146	52.424
PEDERNALES	LGCB	-1.000	-7.288	6.633	93.513
PEDERNALES	SNLR	-1.000	-9.349	5.846	221.367

Table D.3. Details about the data used to produce Figure 3 and Table 1 in the main text. Note that the detection procedure is only applied on the East component. Note that when the detection time is -1, it means that no significant postseismic signal has been detected.
